# Enhancing Veress Needle Entry with Proximal Vibroacoustic Sensing for Automatic Identification of Peritoneum Puncture

**DOI:** 10.3390/diagnostics14151698

**Published:** 2024-08-05

**Authors:** Moritz Spiller, Nazila Esmaeili, Thomas Sühn, Axel Boese, Salmai Turial, Andrew A. Gumbs, Roland Croner, Michael Friebe, Alfredo Illanes

**Affiliations:** 1 SURAG Medical GmbH, 04229 Leipzig, Germany; nazila@surag-medical.com (N.E.); thomas@surag-medical.com (T.S.); alfredo@surag-medical.com (A.I.); 2 Chair for Computer Aided Medical Procedures and Augmented Reality, Technical University of Munich, 85748 Munich, Germany; 3 Department of Orthopaedic Surgery, Otto-von-Guericke University Magdeburg, 39106 Magdeburg, Germany; 4 INKA—Innovation Laboratory for Image Guided Therapy, Otto-von-Guericke University Magdeburg, 39106 Magdeburg, Germany; axel.boese@med.ovgu.de (A.B.); friebe@mac.com (M.F.); 5 Department of Pediatric Surgery and Pediatric Traumatology, University Clinic for General, Visceral, Vascular and Transplant Surgery, University Hospital Magdeburg, 39120 Magdeburg, Germany; salmai.turial@med.ovgu.de; 6 University Clinic for General, Visceral, Vascular and Transplant Surgery, University Hospital Magdeburg, 39120 Magdeburg, Germany; aagumbs@gmail.com (A.A.G.); roland.croner@med.ovgu.de (R.C.); 7 Advanced & Minimally Invasive Surgery Excellence Center, American Hospital Tblisi, 0102 Tblisi, Georgia; 8 Faculty of Computer Science, AGH University of Science and Technology, 30-059 Krakow, Poland; 9 Center for Innovation, Business Development & Entrepreneurship, FOM University of Applied Sciences, 45141 Essen, Germany

**Keywords:** capnoperitoneum, guidance systems, laparoscopy, laparoscopic access, minimally invasive surgery, surgical support, vibroacoustics

## Abstract

Laparoscopic access, a critical yet challenging step in surgical procedures, often leads to complications. Existing systems, such as improved Veress needles and optical trocars, offer limited safety benefits but come with elevated costs. In this study, a prototype of a novel technology for guiding needle interventions based on vibroacoustic signals is evaluated in porcine cadavers. The prototype consistently detected successful abdominal cavity entry in 100% of cases during 193 insertions across eight porcine cadavers. The high signal quality allowed for the precise identification of all Veress needle insertion phases, including peritoneum puncture. The findings suggest that this vibroacoustic-based guidance technology could enhance surgeons’ situational awareness and provide valuable support during laparoscopic access. Unlike existing solutions, this technology does not require sensing elements in the instrument’s tip and remains compatible with medical instruments from various manufacturers.

## 1. Introduction

Establishing laparoscopic access, a critical step in surgical procedures, is prone to complications, with 30–50% occurring during access creation, resulting in an overall complication rate of 14%, including a 4% incidence of intraoperative complications [1,2,3,4,5]. About 30% to 50% of all intestinal injuries and 13% to 50% of all vascular injuries remain undetected during surgery [1], which can lead to sepsis and a mortality rate of 5% to 15% due to this type of injury [6,7,8]. Despite the availability of optical trocars and alternatives, the Veress needle remains widely used, emphasizing the need for improved access methods [6,9,10,11].

Surgeons rely on subjective touch and audible cues during Veress needle insertion, leading to complications such as pre-peritoneal CO_2_ inflation and organ injuries [2,12]. Complications are under-reported, highlighting the necessity for enhanced safety measures [8,10,13,14,15]. Moreover, there is a direct relationship between the surgeons’ experience and their perception of the feedback [12].

To overcome these challenges and improve patient outcomes, the industry and the scientific community have proposed several alternative instruments with added guidance characteristics. Today, surgeons can choose between standard surgical instruments, optical trocars, sensor-based solutions, and tracking systems for establishing laparoscopic access. In the following, the most important solutions are described.

Despite their known drawbacks, standard Veress needles and trocars are still widely used for establishing laparoscopic access and are considered the gold standard in clinical practice. These instruments are simple to use, low in cost, and commonly trained in surgical education. However, their use imposes significant risks as described above and frequently leads to intra- and postoperative complications [1,2]. A recent study found that 36% of surgeons using the Veress needle do not feel safe and perceive a risk of injuring the patient during laparoscopic access. Additionally, 55% reported that the Veress needle’s spring mechanism does not clearly indicate the reaching of the peritoneal cavity [16].

Improved trocars and Veress needles with added guidance aim to address complications associated with standard Veress needles and trocars, which contribute to 30–50% of laparoscopic procedure issues. Optical trocars, offered by companies like Karl Storz, Applied Medical, Medtronic, and Ethicon, provide real-time imaging during tissue layer transection using an inserted endoscope. Modified Veress needles like LaparoLight (ConMed) and EpiAccess (EpiEP) offer visual placement indicators, with LaparoLight utilizing a light at the proximal end to signal entry. Another patented approach by Enable Inc. incorporates a miniaturized camera into a modified Veress needle, though it is not commercially available.

However, these solutions present drawbacks, such as an increased cost, limited guidance, and low versatility, making them non-standard and less prevalent in clinical practice. Studies, including one by Bhoyrul et al. [17], highlight complications despite trocars with ’safety shields,’ with FDA reports underscoring serious issues related to optical trocars. The term ’safety shields’ is prohibited in optical trocar product labeling due to their ineffectiveness in protecting intra-abdominal organs and blood vessels, as indicated by 79 serious complications reported by the FDA [18,19,20].

Despite various sensor-based solutions proposed for laparoscopic access, none have gained traction in regular clinical practice. Examples include an adapted trocar with pressure, accelerometer, and impedance sensors, aiming to differentiate tissues upon contact [21]. Fontanelli et al. introduced a force-based sensing system integrated into a trocar for robotic surgery, evaluated in a 3D-printed prototype without real-world application [22]. Another study employed a three-degrees-of-freedom force sensor on the trocar, estimating forces with less than 6.30% error but not progressing further [23].

Postema et al. proposed a safety mechanism for the Veress needle, reducing the risk of overshooting by decoupling the surgeon’s hand after entering the abdomen. The prototype, tested in an ex vivo porcine model, demonstrated a 50% reduction in overshooting but necessitates Veress needle design changes and re-certification as a medical product [24].

In summary, these proposed solutions involve novel tool developments, potentially incurring elevated costs and modifications to clinical workflows. Their benefits, including incremental enhancements to existing instrumentation, show minimal improvement in safety compared to traditional instruments. Consequently, the conventional use of Veress needles and trocars persists among surgeons, emphasizing the need for a paradigm-shifting approach to enhance precision, safety, and efficiency in laparoscopic access.

The Surgical Audio Guidance (SURAG) sensing concept is an innovative technology with high potential for guiding needles in minimally invasive procedures. It utilizes vibroacoustic (VA) waves generated by the needle’s tip interacting with tissue, detected by a module at the proximal end. These signals are processed to provide interpretable feedback during surgery. SURAG can automatically identify and magnify tissue–tissue passages and punctures of layers like the fascia or peritoneum, offering visual and/or acoustic feedback. It is a plug-and-play system compatible with existing Veress needles and trocars, and is significantly more cost-efficient than optical trocars [25].

Preliminary proof with a standard biopsy needle revealed valuable dynamical information in the VA signal, correlating with the force exerted for needle insertion [25]. Recent assessments using ex vivo animal tissue and a synthetic model involved over a thousand Veress needle insertions, generating an extensive audio signal dataset. The signals were analyzed to magnify and distinguish key puncture events, particularly when penetrating crucial layers like the fascia and peritoneum. Certain characteristics extracted from the VA signals allow for the automated categorization of punctures, addressing the crucial need for clear indicators, as emphasized by experienced laparoscopic surgeons [16,26,27]. This underscores SURAG’s potential to enhance laparoscopic access.

This article presents the results of porcine cadaver experiments. During the experiments, a prototype, called EasyAccess, of the SURAG sensing concept was used. The aim of this study is twofold. First, it seeks to assess whether the findings observed in phantom studies hold true for needle insertions into the porcine cadaver’s abdomen. This evaluation aims to analyze and understand the signal dynamics of a peritoneum puncture event and determine whether the VA excitation resulting from this puncture exhibits signal signatures suitable for identifying a puncture event as a cavity entry. Second, the study aims to explore the different signal phases involved during the puncture of the peritoneum and their relationship in terms of signal energies and temporal durations.

The experiments not only validate the applicability of previously observed vibroacoustic phenomena in real porcine cadaver abdomen scenarios but also enhance the understanding of these dynamics during peritoneum puncture. Through the use of time–frequency representations and transient excitation pattern identification, the occurrence of cavity puncture events could be confirmed. These findings suggest that the SURAG sensing concept is the first approach achieving event identification and characterization using existing standard instruments and non-invasive sensing as the device is connected to the proximal end of the instrument. This could potentially enhance precision and safety during laparoscopic access and other minimally invasive procedures.

## 2. Materials and Methods

In the following, the experimental setup, data acquisition, and data analysis of the porcine cadaver experiments are described.

### 2.1. Experimental Setup and Data Acquisition

As described in Section 1, the SURAG sensing concept is based on the acquisition of VA signals, resulting from tool–tissue interactions. The SURAG sensing concept was implemented in EasyAccess, a prototypical system specifically designed for needle applications. In this study, EasyAccess consisted of a sensing unit that was used along with a Lenovo notebook (Lenovo T490) for processing and real-time display. The sensing unit’s housing was designed with a female Luer-lock adapter allowing it to be mounted easily to the standard Luer-lock of a 150 mm single-use Veress needle (Applied Medical, Rancho Santa Margarita, CA, USA) at its proximal end. In this way, the Veress needle remains unaltered while the Luer-lock connection ensures the reliable transmission of vibroacoustic signals.

**Figure 1:**
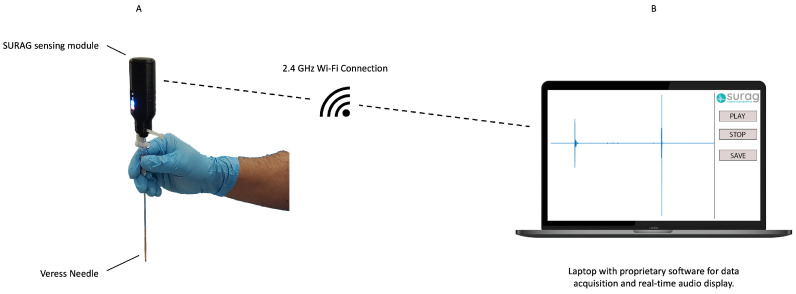
SURAG EasyAccess consists of (**A**) the sensing unit, proximally mounted to the Veress needle via the Luer-lock connector for recording of vibroacoustic signals, and (**B**) a laptop running a proprietary SURAG application for storage and visual and acoustic real-time display. Both components are connected via a 2.4 GHz wireless local area network.

The sensing unit was designed by the authors and consists of a custom printed circuited board (PCB), with buttons, LEDs, and a piezo sensing element, mounted to a Raspberry Pi Zero W (Raspberry Pi Foundation, Cambridge, UK). The sensing unit acquires the VA signals during Veress needle insertion and transmits them via a wireless local area network (WLAN) to an off-the-shelf laptop. The laptop runs a proprietary software application built in Python 3.6. The application receives the signal from the sensing unit and stores it in WAVE file format at a sampling frequency of 16 kHz in the laptop’s internal hard disk. To be able to monitor the insertion, a proprietary software running on the laptop provided real-time acoustic and visual feedback of the VA signal. The visual feedback consists of the real-time display of the VA signal, and the acoustic feedback provides the real-time processed audio that magnifies the main needle tip–tissue interactions. The SURAG EasyAccess prototype used in this study is shown in Figure 1.

**Figure 2:**
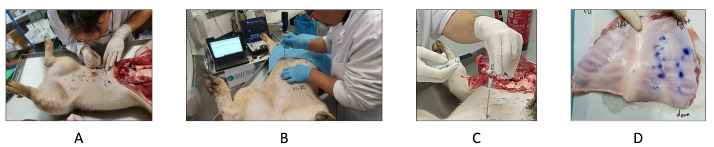
This figure displays the steps for Veress needle insertion and obtaining ground truth during data acquisition. (**A**) The intended insertion points were marked on the abdominal wall before (**B**) Veress needle insertion. (**C**) The commonly used hanging drop test was performed using ink. In that way, successful Veress needle insertions were to be marked on the inside of the abdominal wall. (**D**) After all insertions were performed, the abdominal wall was removed and successful Veress needle insertions could be recorded using the inked insertions points.

Figure 2 shows the implemented experimental setup used to acquire the audio signals from the Veress needle. Eight fresh porcine cadavers weighing from 30 to 70 kg were used for the experiments. Porcine cadavers were selected to model the human abdominal wall because of its analogous underlying anatomy and similar mechanical properties to humans [28].

In each experiment, the porcine cadaver was placed supine on the operating table. After cleaning the abdominal area, the locations for Veress needle insertion were marked on the porcine cadaver’s abdomen. These locations were defined according to the main entry points for inserting the Veress needle in humans, including the sub-umbilicus, Lee–Huang, and Palmer’s point locations [29] (see Figure 2A).

At each location, after making a 5 mm skin incision, a Veress needle was slowly inserted at a $90^{\circ }$ angle relative to the abdomen. This approach is in line with current standard procedures for creating laparoscopic access. The insertion was performed by medical students who were undergoing laparoscopic training at the time of the experiments. Before inserting the Veress needle (see Figure 2B), the sensing module was attached to its proximal end (compare to Section 2.1). After five insertions, the needle was replaced by a new one to ensure an insertion with a sharp needle tip.

Two different verification tests were performed to confirm the correct placement of the Veress needle. The first test was carried out based on the saline drop test approach immediately after the needle was inserted (see Figure 2C). The saline drop test is a standard test used to confirm the position of the inserted Veress needle into the abdomen [30]. For that, a few drops of saline liquid are poured into the Veress needle, and the liquid flow is observed. If the liquid passes through the needle and disappears immediately, it confirms that the needle reached the peritoneal cavity. In the current study, this test was performed using blue ink instead of saline liquid to mark the exit location of the Veress needle on the other side of the abdominal wall. This approach was used as the second verification test (see Figure 2D). After finalizing the last Veress needle insertion, the abdominal wall was removed, especially the region around the insertion locations. Each exit location of the Veress needle was evaluated visually to confirm the passage of the needle through the peritoneum.

A total of 193 VA signals were acquired from a cohort of eight deceased porcine subjects. To enhance the precision and depth of analysis, a video camera was seamlessly incorporated into the experimental setup, enabling the synchronous recording of the entire experimental procedure. This simultaneous video capture was meticulously synchronized with the VA signals, facilitating the identification of peritoneum puncture events during Veress needle insertions.

Time instants of peritoneum puncture were carefully identified through manual annotation applied to each recording. From the experimental sessions on each porcine cadaver, video recordings of successful insertions reaching the peritoneal cavity were chosen for further analysis of the VA signals. In this process, an experienced professional familiar with medical data processing systematically observed and noted the time points of peritoneum puncture events. These annotations were made precisely when the person inserting the needle confirmed the loss of resistance during needle placement. These marked time instants became crucial reference points for subsequent phases of comprehensive VA signal processing and analysis.

### 2.2. Data Analysis

**Figure 3:**
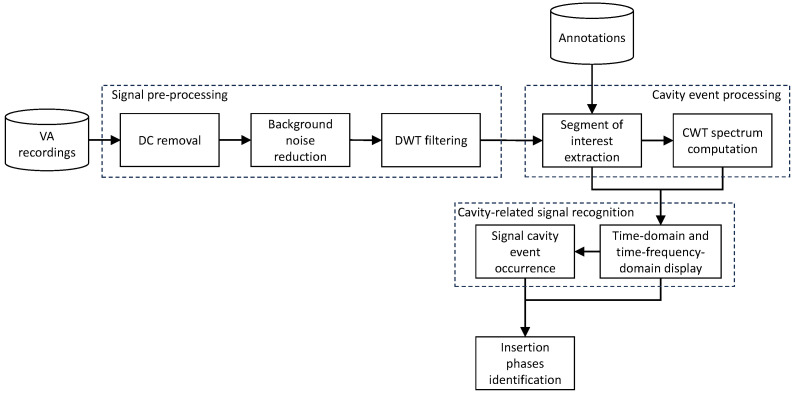
For data analysis, the VA data were processed in multiple steps. The pre-processing steps included DC removal, background noise reduction, and DWT filtering. To obtain signal segments that contain peritoneum punctures, the VA signals were manually annotated, the segment of interest was extracted, and the segment’s continuous wavelet transformation (CWT) spectrum was computed. Based on the time-domain and time–frequency-domain display, the specific insertion phases could be identified.

Figure 3 presents a comprehensive block diagram outlining the methodology employed for data analysis, which is composed of four primary steps. The initial step encompassed pre-processing procedures designed to mitigate noise and amplify signal dynamics related to needle tip–tissue interactions. The second phase aimed to locate and extract the region of interest around the annotation of cavity events, subsequently computing their time–frequency characteristics. In the third stage, a thorough investigation of the signal segment and its corresponding time–frequency spectrum was conducted to confirm the presence of cavity puncture events. The final step involved the identification of key phases within a cavity puncture by identifying dynamical changes in the signal.

Before detailing each step of the algorithm, it is important to understand the main characteristics of the vibroacoustic signals resulting from interactions between the Veress needle tip and the tissue. Each interaction results in fast transient variations with multiple frequency changes within an extremely short period. The main difference between a cavity puncture and other interaction events is that tissue–tissue transitions produce transient responses that are rapidly damped by the subsequent tissue. This contrasts with the peritoneum puncture, where, because it is a cavity, the needle experiences a free excitation rather than damping. These fast transient dynamical changes, involving several frequency shifts, cannot be effectively tracked using standard Fourier-based techniques. This is why we use wavelet transformation in this work, as it is well suited for capturing these highly transient characteristics. Specifically, discrete wavelet transformation (DWT) is employed to enhance the transient characteristics, while continuous wavelet transformation (CWT) is used to track the fast dynamical changes within the transient response.

The core objective of the signal pre-processing phase was to diminish various noise types while enhancing events related to needle tip–tissue interactions. Initial steps included DC removal, followed by background noise reduction through a spectral gating technique. This involved computing the signal’s spectrogram and establishing a frequency-dependent noise threshold (gate) for each frequency band of the signal/noise ratio [31]. The resultant threshold was then utilized to generate a mask that effectively attenuated noise below the threshold in a frequency-dependent manner. Subsequently, the signal was filtered to reduce low-frequency baseline trends and emphasize high-frequency components known to relate directly to needle tip–tissue interaction dynamics. For that, a discrete wavelet transformation (DWT)-based filter was employed. The signal was decomposed into ten scales using a Daubechies discrete wavelet transformation (DWT) and then reconstructed with selected frequency wavelet scales [32].

In the second step, a segment of interest was delineated around the annotated time instant of the cavity event. A 400 ms window was generated around this time instant, and the signal was extracted within this window. Considering the transient attributes of puncture event signals, the continuous wavelet transformation (CWT) was computed over the signal segment, serving as its time–frequency spectrum representation.

To confirm the detectability of cavity punctures, the third step involved the visualization and analysis of time-domain and time–frequency representations of the signal segment. This process was carried out to identify significant transient excitation patterns within both the signal and its spectrum, thereby determining the occurrence or absence of a cavity puncture event.

Finally, in the fourth step, upon confirming a cavity event within the signal segment, the distinct dynamical changes evident in both the time domain and time–frequency domain of the signal segment were used to categorize various phases occurring during a needle puncture event.

## 3. Results

### 3.1. Qualitative Results

This section presents qualitative outcomes derived from cavity puncture events. The main goal is to analyze and understand the signal dynamics of a cavity event and how they are related with the insertion’s physical process.

**Figure 4:**
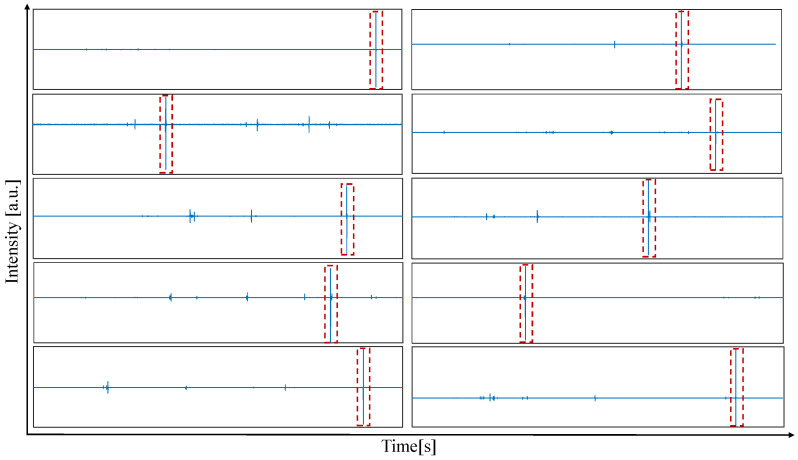
The entry of the Veress needle into the peritoneal cavity (marked in red frames) can clearly be observed in the time-domain vibroacoustic signal. This figure displays ten exemplary signals; however, this was observed in 100% of the acquired signals.

Figure 4 illustrates time-domain VA signals from ten distinct recordings captured during the full Veress needle insertion process. It can be observed that the signal excitations of a peritoneum puncture are significantly higher than the excitations of other puncture events, such as a puncture to a fascia. This observation can be explained by the interaction between the needle and the surrounding tissue. When a tissue–tissue puncture occurs, for example, a puncture of a fascia, the energy induced by the release of the spring mechanism is damped by the second tissue layer, leading to damping. When a peritoneum puncture occurs, i.e., a tissue-cavity puncture, this damping is only minimal, leading to a signal intensity that clearly dominates other events. This behavior facilitates the accurate identification of a peritoneum puncture.

**Figure 5:**
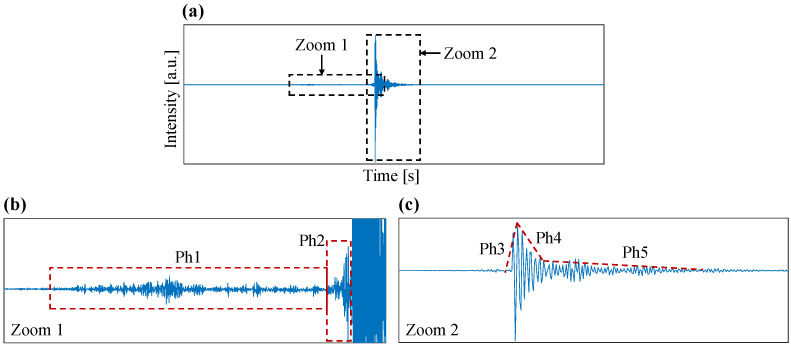
The VA signal obtained (blue line) during Veress needle insertion (**a**) can be divided into two segments for closer examination. The pre-puncture phase consists of the friction between inner and outer core of the Veress needle and the actual puncture to the peritoneum, including tissue breakage (**b**). The post-puncture phase is characterized by the click sound of the Veress needle caused by the release of the needle’s spring mechanism (**c**). The phases are separated by the red dot lines in the figure.

Upon closer examination of the excitation event through a zoomed view (Figure 5), distinct signal characteristics, corresponding to various phases during the needle insertion process, could be identified. To enhance clarity in visualizing these characteristics, we divided the zoomed signal into two segments (Figure 5a). The initial segment of the signal (Figure 5b) reveals dynamic features related to friction occurring between the inner and outer cores of the needle (phase Ph1) and the puncture of the peritoneum, where tissue breakage occurs just before entering the peritoneal cavity (phase Ph2). In the subsequent segment of the signal (Figure 5c), we distinguish phases associated with the excitation of the click sound. This includes a rapid increase (attack) in the energy of the excitation resulting from the click sound (Phase Ph3) and a recovery dynamic involving a fast recovery phase (Phase Ph4) followed by a slower recovery phase (Phase Ph5). These phases originate from the release of tension within the spring mechanism and the subsequent vibration of the needle structure.

The CWT time–frequency spectrum of the zoomed excitation reveals the dynamic signature of the vibroacoustic (VA) signal during each insertion phase.

**Figure 6:**
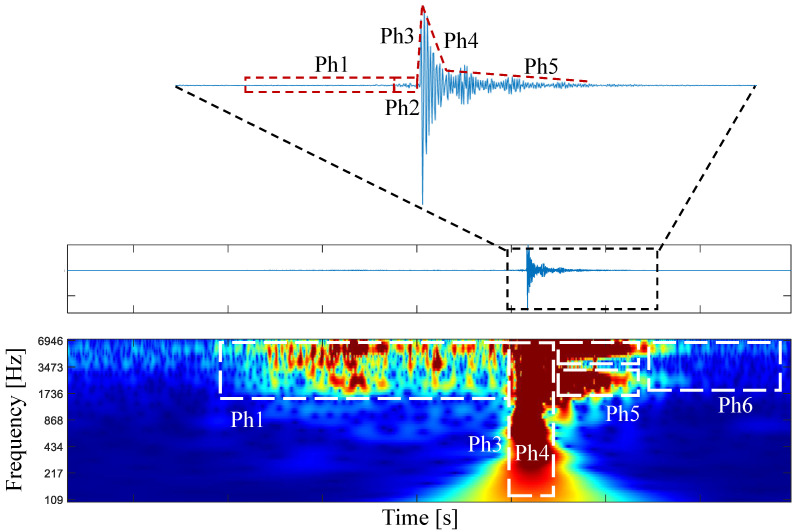
CWT time–frequency spectrum representation of a cavity event, where it is possible to observe the spectral component characteristics of each cavity event phase next to the original signal (blue line at the top of the figure). In this depiction, the tissue breakage (Phase 2) is hidden in the time–frequency due to the high energy of the release of the needle’s spring mechanism (Phase 3) and is therefore not marked here. The phases are separated by the red and white dot lines.

As depicted in Figure 6, the main excitation dynamics and phases manifest as distinct signatures in the time–frequency domain. Notably, the dynamics of friction mainly occur within the frequency range of 1800 Hz to 7000 Hz. As illustrated in Figure 6, the main excitation dynamics and phases are clearly evident as distinct patterns in the time–frequency domain. Notably, the friction dynamics primarily occur within the frequency range of 1800 Hz to 7000 Hz. Due to the transient nature of the click dynamics, the frequency response spans the entire spectrum. The recovery phase exhibits frequencies similar to those of the friction phase but with a distinctly different time–frequency signature. Even after the needle excitation ends, tiny signal variations persist as the needle takes time to reach a stable state. This corresponds to the post-puncture dynamics (Phase Ph6).

In summary, building upon the analyses conducted in both the time domain and time–frequency domain, six distinct signal phases emerge during a cavity puncture event:

- Phase 1 (Ph1): Friction between the needle’s inner and outer cores.
- Phase 2 (Ph2): Needle puncture involving the breaking of the peritoneum tissue.
- Phase 3 (Ph3): Attack phase during the release of spring tension.
- Phase 4 (Ph4): Decline phase following the release of spring tension.
- Phase 5 (Ph5): Release phase after the spring tension is released.
- Phase 6 (Ph6): Post-puncture phase characterized by the cessation of needle vibration and a return to a new stationary stage.

### 3.2. Quantitative Results

Table 1 presents the comprehensive dataset of Veress needle insertion experiments conducted across eight porcine cadavers. The first three columns detail the total count of needle insertions per porcine cadaver, along with the count of insertions where successful arriving into the cavity was achieved. The remaining four columns illustrate the occurrence of audio excitations, as well as the most important excitation phases analyzed in Section 3.1, assessed within the experiments where the cavity was reached. It is notable that a clear excitation is evident in the resulting VA signal in 100% of cases where cavity penetration was achieved. Furthermore, within all VA excitations, the identification of the inner–outer core friction (Phase 1) is consistently identifiable. The precise moment of effective peritoneum tissue breakage or puncture was discernible in 141 out of 154 cavity puncture events (91.55%). As anticipated, Phase 3, corresponding to the spring release, was identifiable in 100% of cavity puncture events.

**Figure 7:**
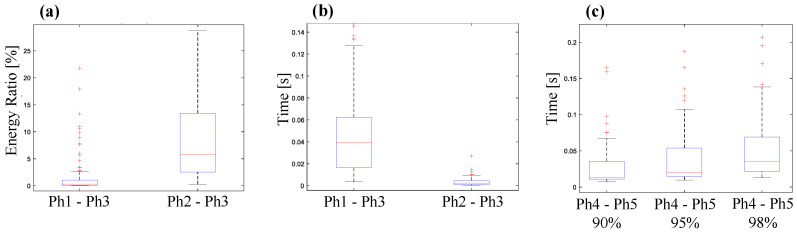
Boxplots showing the statistics of the full set of experiments for (**a**) the extracted energy ratio features between friction and puncture events compared to click sound, (**b**) time duration of inner–outer core friction and latency between puncture and click sound, and (**c**) recovery phase duration after spring tension release considering 90%, 95%, and 98% of the event energy. The red line represents the median value, while the blue boxes enclose the IQR (Interquartile Range). The black dot lines represent the minimum and maximum values, while the red pluses display outliers.

Figure 7 highlights results involving the distinctive characteristics of key needle insertion phases. The objective is to elucidate the dynamical relations between the needle insertion phases, considering both energy levels and associated time intervals.

In Figure 7a, a boxplot depicts the energy ratios, expressed as percentages, between the friction and puncture phases (Phase 1 and Phase 2) in comparison to the click sound phase (Phase 3). This analysis is based on the complete dataset of experiments where these phases are discernible. The energies for each phase were computed by summing the squares of the signal segments corresponding to the given phase. As anticipated, it is evident that Phase 3 exhibits a significantly higher energy level compared to Phase 1 and Phase 2. Specifically, the peak energy of a click sound excitation can surpass that of peritoneum puncture (Phase 2) by approximately 20 times and exceed the energy of inner–outer core friction (Phase 1) by around 200 times. While the click sound’s excitation amplitude can be substantial enough to potentially overshadow other significant dynamics, it is important to note that both Phase 1 and Phase 2 remain distinctly recognizable and audibly discernible within the VA signal.

Figure 7b presents time intervals or latencies for each experiment in the form of a boxplot. On the right side of the figure, the duration of the friction phase (Phase 1) is depicted, while, on the left side, the latency between the moment of peritoneum tissue breakage (peritoneum puncture, Phase 2) and the instant when spring tension is released is illustrated. The median duration of the inner–outer core friction phase is approximately 20 ms, with a considerable dispersion of over 20 ms. This variability can be attributed to the dependence of Phase 1 on the insertion velocity of the needle. The latency between Phase 2 and Phase 3 is notably brief, as the spring tension release immediately follows peritoneum puncture. This latency measures less than 5 ms and exhibits minimal dispersion as it depends solely on internal mechanical characteristics of the needle.

Finally, Figure 7c illustrates the duration of the recovery time during Phase 4 and Phase 5. The recovery time is computed by thresholding the instantaneous energy ratio from the peak of the excitation until the end of the excitation. When the instantaneous energy ratio falls below a threshold, represented as a percentage of the total event energy, this time is considered as the end of the recovery phase. We present the recovery time results at three thresholds: 90%, 95%, and 98% of the total recovery event. In the first scenario (90% of the energy), the recovery phase exhibits a median duration of 13 ms. When considering the scenario where energy is computed until reaching 95%, the recovery phase extends to a median of 20 ms. In the final scenario, encompassing 98% of the energy, the recovery phase lasts for 35 ms. These results highlight the short duration of the puncture excitation recovery phase. Furthermore, considering the entire puncture event from the initiation of inner–outer core friction, its median duration is less than 80 ms.

## 4. Discussion

This study aimed to assess if the results observed during Veress needle insertion into phantoms also hold true for Veress needle insertions into a porcine cadaver’s abdomen and if the puncture of the peritoneal cavity could be identified in the VA excitation of the needle. Furthermore, the study explored the connections between distinct dynamics that arise during peritoneum puncture.

The results of this study reveal that each phase of a puncture to a main tissue layer can be clearly identified in the signal. This includes the friction between the needle’s inner and outer core, which occurs before every puncture, but also the puncture itself and the release of the needle’s spring mechanism. It could also be observed that a puncture event lasts less than 100 ms, which makes it very difficult to be perceived by humans. However, since the SURAG sensing concept not only provides access to the puncture itself but also to dynamical processes that occur upfront, the system could be used for making entry into the peritoneal cavity more perceivable by expanding the puncture event and displaying it as acoustic feedback to surgeons. In previous studies, acoustic feedback has been evaluated and found beneficial for supporting surgical interventions [33,34,35]. Worth highlighting is also the observation that tissue cavity punctures induce significantly different signal dynamics than other puncture events. This suggests that the SURAG sensing concept could also provide navigation support during other needle placement tasks that involve the arrival of the needles tip to a cavity, such as spinal anesthesia, lumbar puncture, or thoracentesis.

During the analysis, it was also observed that dynamical processes lasting less than 5 ms could be detected in the signal. Presumably, this makes the SURAG technology a powerful and cost-efficient tool for monitoring extremely rapid processes in manufacturing, quality assurance, and other sectors in real time.

Quantitative analysis of the acquired signals also revealed that the friction (Phase 1), as well as the release (Phase 3), could be identified in 100% of the cases, while the puncture of the peritoneum layer (Phase 2) was identified in 91.55% of the insertions. Since the entry into the peritoneal cavity is always characterized by the temporal order of friction (Phase 1), puncture (Phase 2), and release (Phase 3), this indicates that the entry into the peritoneal cavity can be reliably identified using EasyAccess. While punctures exclusively characterize entries into the peritoneal cavity, friction could also be caused by other tissue structures, such as (muscle) fibers. Further studies need to be performed to evaluate if friction caused by the entry to the peritoneal cavity and friction artifacts could be distinguished from each other, potentially by employing machine learning [27]. While this would improve manual laparoscopic access, it would also provide a basis for autonomous needle insertions in surgery as described in [36].

However, the road to autonomous surgery will be a long process with multiple steps [36]. Because of the relatively low amount of data obtained during VA sensing, the SURAG sensing concept may be an interesting route to more intelligent procedures in the future. Since modern surgery encompasses not only traditional methods such as laparoscopic and robot-assisted surgery but also more complex procedures (e.g., interventional radiology), it may be more prudent to focus on autonomous actions during percutaneous procedures, such as Veress needle insertion, prior to embarking on more complex tasks, such as autonomous actions during laparoscopic and robotic-assisted surgery. In the future, AI-based systems could play a significant role in supporting surgical procedures and decision making [37]. Moreover, the SURAG approach may represent the first step in a brand-new way to obtain haptics during robotic surgery. The SURAG system may enable the computer to effectively obtain audiohaptics, which may be imperceptible to the human but may currently be the ideal way for surgical robots to safely navigate the human body [38].

Previously discussed alternative solutions for laparoscopic access outlined in Section 1 exhibit shared limitations, notably their proprietary nature, single-use design, and high cost. In some instances, these alternatives incorporate electronic components within the instrument’s shaft and tip, potentially elevating patient risk. Conversely, SURAG presents a distinct approach. Operating as an add-on device to existing instruments, it introduces enhanced cost-effectiveness and user-friendliness. All its constituent parts are positioned at the proximal end of the instrument, thus contributing to increased patient safety and streamlining the certification process.

Like the alternative solutions introduced above, SURAG EasyAccess is not a predictive device that can prevent injuries, such as intestinal punctures, before they occur. However, it has the potential to notify users of such injuries, which would be a significant advancement since intestinal injuries often go unnoticed [1]. Additionally, the high sensitivity of the sensing unit results in the acquisition of a large amount of irrelevant data. This necessitates increased processing and, for applications other than detecting peritoneum punctures, could reduce accuracy in clinical use.

Established methods for confirming successful Veress needle placement have reported accuracies ranging from 60% [30] to 96% [39]. These figures are notably lower than the proportion of insertions in this study where peritoneum entry was identifiable through vibroacoustic signals. Furthermore, the unique signal characteristics associated with the peritoneum puncture by a Veress needle (refer to Table 1) were present in approximately 95% of the cases. Although a direct comparison with the accuracies of traditional methods is not feasible, these findings underscore the potential of this technology to offer highly accurate detection of the Veress needle’s arrival into the peritoneal cavity.

In summary, the findings of this study confirmed that the SURAG sensing concept can serve for automatically identifying Veress needle entry into the peritoneal cavity based on its acquired vibroacoustic signals. This further strengthens the assumption that the technology could be a cost-efficient, simple, and safe means to provide real-time insights and monitor Veress needle insertion, making laparoscopic access more precise, safe, and efficient.

## 5. Conclusions

This article presented the results of porcine cadaver experiments using SURAG EasyAccess that were conducted to assess the applicability of previous phantom experiments to more realistic conditions. The results demonstrate that each peritoneum puncture produces a clear excitation with distinguishable patterns. This information lays the groundwork for future classification efforts. In line with the results of the phantom experiments, this work led to the following research findings:

1. The vibroacoustic signals acquired by EasyAccess allow for the **exact identification** of all phases of a Veress needle insertion including the peritoneum puncture.
2. Based on Item 1 above, it can be concluded that the acquired signal can be processed and **transformed into, e.g., acoustic feedback that enhances the situational awareness of surgeons**.
3. The ability to identify the particular phases of a needle insertion (see Item 1 above) indicates that EasyAccess could also serve for the **automatic, artificial Intelligence (AI)-based identification of needle insertion events,** such as the peritoneum puncture.

We estimate that a medical product based on the prototype used in this study could be supplied to hospitals for under EUR 30, making it significantly more cost-effective than current solutions. This affordability is achieved through the use of cost-efficient vibroacoustic sensors and by maintaining the existing medical instrument as unaltered. This approach not only supports broader access to advanced medical treatment but also ensures ease of use, as healthcare professionals can continue using their familiar instruments and stick to their existing workflows.

The performance of the vibroacoustic sensing method was evaluated using a single-use Veress needle in this study. It is noteworthy that all Veress needles available in the market are equipped with a Luer-lock connector at the proximal end, facilitating the easy attachment of the sensing module. The materials used in disposable Veress needles from various manufacturers are generally similar, which helps to minimize potential signal variations. However, the necessity to validate these findings across different needle models is recognized. As a result, future experiments will involve testing with a minimum of three different disposable Veress needle models. This approach will ensure the robustness and generalizability of the method. It will also help in confirming that the identified parametric phases and free excitation puncture characteristics remain consistent across different types of needles.

This study reveals that each peritoneum puncture event generates a unique vibroacoustic signal excitation, observed in all cases. These distinct characteristics can be parameterized for event classification using machine learning techniques, marking an exciting direction for future research. We plan to validate these findings in human cadavers and conduct first-in-human studies. A machine learning model will be developed using selected features from our data for testing in these settings. In summary, the findings suggest that vibroacoustics could be a powerful tool to enhance the safety and efficacy of laparoscopic access and further needle information.

## Figures and Tables

**Table 1:** Summary of Veress needle insertion experiments and excitation phases. The data highlight the consistent identification of excitation phases, such as inner–outer core friction (Phase 1), peritoneum tissue breakage (Phase 2), and spring release (Phase 3).

Cadaver	Number of Needle Insertions	Number of Needle Insertions Reaching Peritoneal Cavity	Number of Audio Excitations Resulting from Cavity Puncture	Number of Cavity Audio Excitations with the Presence of Phase 1	Number of Cavity Audio Excitations with the Presence of Phase 2	Number of Cavity Audio Excitations with the Presence of Phase 3
Cadaver01	13	10	10	10	10	10
Cadaver02	20	16	16	16	14	16
Cadaver03	15	11	11	11	9	11
Cadaver04	30	23	23	23	19	23
Cadaver05	21	19	19	19	18	19
Cadaver06	21	18	18	18	17	18
Cadaver07	45	34	34	34	32	34
Cadaver08	28	23	23	23	22	23
Total	193	154	154	154	141	154

## Data Availability

The data presented in this study are available on request from the corresponding author. The data are not publicly available due to specific regulations and policies within the organizations involved in this study.

## Abbreviations

The following abbreviations are used in this manuscript:

AI	Artificial Intelligence
CWT	Continuous Wavelet Transformation
DWT	Discrete Wavelet Transformation
FDA	U.S. Food and Drug Administration
SURAG	Surgical Audio Guidance
US	Ultrasound
VA	Vibroacoustic
WLAN	Wireless Local Area Network
